# Complete Genome Sequence of Sacbrood Virus Strain SBM2, Isolated from the Honeybee *Apis cerana* in Vietnam

**DOI:** 10.1128/genomeA.00076-12

**Published:** 2013-01-31

**Authors:** Nga Thi Bich Nguyen, Thanh Hoa Le

**Affiliations:** Immunology Department, Institute of Biotechnology, Vietnam Academy of Science and Technology (VAST), Hanoi, Vietnam

## Abstract

Here we report the complete genomic sequence of the SBM2 strain (VSBV-SBM2) of the sacbrood virus (SBV) that was isolated from the Asian honeybee (*Apis cerana*) in Northern Vietnam. The entire sequence excluding the 3′ poly(A) tail is 8,834 nucleotides in length and contains a single large open reading frame (ORF) of 8,580 nucleotides (position 178 to 8757), encoding 2,859 amino acids. VSBV-SBM2 shared 90 to 93% nucleotide identity and 95 to 96% amino acid homology to six complete genomes of SBV currently available in GenBank (two from China, three from Korea, and one from the United Kingdom). A hypervariable domain (amino acid [aa] position 712 to 729) and a conserved motif (position 2124 to 2143) in the precursor polypeptide of all seven SBVs are also described.

## GENOME ANNOUNCEMENT

The sacbrood virus (SBV) primarily infects broods of both common European (*Apis mellifera*) and Asian (*Apis cerana*) honeybee species, causing larva death due to failure of pupation that leads to the formation of a sac-like shape. This picorna-like virus belongs to the genus *Iflavirus* in the family *Iflaviridae* and contains a single-stranded positive-sense RNA genome of about 8.8 kb (1). To date, six complete genome sequences of SBVs are available: strain Rothamstead (GenBank: AF092924) from the United Kingdom; AmSBV-Kor19 (JQ390592), AmSBV-Kor21 (JQ390591), and AcSBV-Kor (HQ322114) from South Korea; and CSBV-LN (HM237361) and CSBV-GZ (AF469603) from China (1–3).

In Vietnam, sacbrood virus infection was first recognized in 2003, with severe infections in both indigenous *A. cerana* and imported *A. mellifera* colonies, and molecular characterization/detection of Vietnam SBVs were carried out and reported in local journals (4, 5). The SBM2 strain of SBV was isolated from an infected sac from an *A. cerana* beehive in a mountainous province in Vietnam. The genomic RNA of the VSBV-SBM2 isolate was extracted from the infected “gondola-like” sac and used as a template for complementary DNA conversion by using random hexamer primers (Fermentas Inc.). Ten overlapping fragments were obtained by PCR using five pairs of oligonucleotide primers designed based on the alignment of the full-length genome sequences of the available SBV strains listed above. PCR products were purified using an AccuPrep gel purification kit (Bioneer, Daejeon, South Korea) and directly sequenced with an ABI 3100 genetic analyzer automated sequencer (Applied Biosystems). The entire genomic sequence was obtained by assembling the overlapping sequences using AssemblyLIGN v 1.0, analyzed by MacVector 8.2 software (Accelrys Inc.). Multiple sequence alignment of the whole genome and the open reading frame (ORF) sequences was performed by GENEDOC 2.7 (6), and the phylogenetic tree analysis was performed using the MEGA5.1 program (7).

The complete genome of SBV strain SBM2 consists of 8,834 nucleotides (nt) in which a 177-nt region of the 5′ untranslated region (5′ UTR) and a 3′ UTR of 77 nt flank a large ORF of 8,580 nt, encoding a polyprotein precursor of 2,859 amino acids. Sequence identity of the SBM2 isolate compared to 6 SBVs from GenBank at the nucleotide level ranged from 90 to 93% for the complete genome, 90 to 94% for ORF, and 95 to 96% for the deduced amino acids. The polyprotein of VSBV-SMB2 has a hypervariable section with a full 17  amino acids (aa) (position 712 to 729), similar to CSBV-GZ of China (8) and AmSBV-Kor21 of South Korea (3). This amino acid sequence is partially deleted in CSBV-LN (13 aa) (2) and in Rothamstead (1 aa) (1) but is completely absent in the AmSBV-Kor19 and AcSBV-Kor strains (3). An identical motif (TACGIYGTVALLPRHYVRA) (position 2124 to 2143) is present in all Vietnamese and Korean strains but is different in Chinese strains. Phylogenetic analysis indicated that the likely position in VSBV-SBM2 is close to the position in CSBV-LN of China.

### Nucleotide sequence accession number.

The genomic sequence of the sacbrood virus strain SBM2 has been submitted to GenBank (accession number KC007374).
